# 

**Published:** 1886-04

**Authors:** 


Page.

ORIGINAL COMMUNICATIONS:

Insane Hospital Supervision. E. C. Spitzka ........ 289

Pelvic Hæmatocele. W. W. Grant .. 318

EDITORIAL:

The Annual Meeting of the American Medical Association .......... 324

FOREIGN CORRESPONDENCE:

Letter from Chiavari, Italy. A. Lagorio ........ 327

SOCIETY REPORTS:

*Chicago Gynæcological Society, February 9th* Laparotomy for Pelvic Abscess. A. R. Jackson ........ 330

A Feeding-Bottle for use in cases of Intubation of the Larynx. F. E. Waxham ........ 351

*Chicago Medical Society, February 1st.* Quinsy as a Rheumatism. J. S. Knox ........ 352

Intubation of the Larynx for acute Catarrhal Laryngitis. A. B. Strong. 353

*Meeting February 15th.* Digital Exploration of the Bladder in the Male. W. T. Belfield ........ 363

Production and Prevention of Laceration of the Perineum. H. T. Byford ........ 371

A Gase of Oöphorectomy and of Nephrectomy. R. G. Bogue ........ 372

Battey's Operation, etc. C. T. Parkes 373

Ossification of the Choroid. W. F. Coleman ........ 376

PAGE.

LOCAL NEWS:

Rush Medical College ........ 378

College of Physicians and Surgeons . 378

Chicago Medical College, University Day ........ 378

Chicago Medical College ........ 379

Cook County Hospital ........ 379

Hospital Appointments ........ 380

ITEMS:

St. Paul Medical School ........ 381

*The Neurological Review* ........ 381

*The Medical Analectic* ........ 382

Insanity from Cataract Operations ... 383

ANNOUNCEMENTS:

The American Medical Association .. 383

The American Surgical Association .. 384

The Illinois State Medical Society ... 384

BOOK REVIEWS:

The Principles of House-Drainage. J. P. Putnam ........ 385

Epilepsy and other Chronic Convulsive Diseases, etc. W. R. Gowars . 385

Climatology and Mineral Waters of the United States. A. N. Bell .... 386

Essentials of Physics and Chemistry. C. W. Culver ........ 387

A Manual for the Practice of Surgery. Thomas Bryant ........ 387

Inorganic Chemistry. E. Frankland and F. R. Japp ........ 388

Clinical Notes on Uterine Surgery. J. M. Sims ........ 389

Practical Suggestions Respecting Electricity, etc., etc. A. L. Ranney .... 389

Books Received ........ 391

Pamphlets Received ........ 392

